# Rescue therapy after thrombectomy for large vessel occlusion due to underlying atherosclerosis: review of literature

**DOI:** 10.3389/fneur.2023.1181295

**Published:** 2023-06-16

**Authors:** Tigran Khachatryan, Mohammad Shafie, Hermelinda Abcede, Jay Shah, Masaki Nagamine, Justin Granstein, Ichiro Yuki, Kiarash Golshani, Shuichi Suzuki, Wengui Yu

**Affiliations:** ^1^Department of Neurology, University of California, Irvine, Irvine, CA, United States; ^2^Department of Neurological Surgery, University of California, Irvine, Irvine, CA, United States

**Keywords:** rescue therapy, intracranial atherosclerosis, intracranial atherosclerosis stenosis, balloon angioplasty, intracranial stenting, intracranial stenosis, failed thrombectomy, literature review

## Abstract

In this review article, we summarized the current advances in rescue management for reperfusion therapy of acute ischemic stroke from large vessel occlusion due to underlying intracranial atherosclerotic stenosis (ICAS). It is estimated that 24–47% of patients with acute vertebrobasilar artery occlusion have underlying ICAS and superimposed *in situ* thrombosis. These patients have been found to have longer procedure times, lower recanalization rates, higher rates of reocclusion and lower rates of favorable outcomes than patients with embolic occlusion. Here, we discuss the most recent literature regarding the use of glycoprotein IIb/IIIa inhibitors, angioplasty alone, or angioplasty with stenting for rescue therapy in the setting of failed recanalization or instant/imminent reocclusion during thrombectomy. We also present a case of rescue therapy post intravenous tPA and thrombectomy with intra-arterial tirofiban and balloon angioplasty followed by oral dual antiplatelet therapy in a patient with dominant vertebral artery occlusion due to ICAS. Based on the available literature data, we conclude that glycoprotein IIb/IIIa is a reasonably safe and effective rescue therapy for patients who have had a failed thrombectomy or have residual severe intracranial stenosis. Balloon angioplasty and/or stenting may be helpful as a rescue treatment for patients who have had a failed thrombectomy or are at risk of reocclusion. The effectiveness of immediate stenting for residual stenosis after successful thrombectomy is still uncertain. Rescue therapy does not appear to increase the risk of sICH. Randomized controlled trials are warranted to prove the efficacy of rescue therapy.

## Introduction

Intracranial atherosclerotic stenosis (ICAS) is one of the common causes of stroke and accounts for up to 50% of all strokes in certain ethnic groups (1–4). Randomized clinical trials have failed to show the efficacy of intracranial stenting in patients with symptomatic severe ICAS (1, 5–10). Some of the landmark studies, including the Stenting and Aggressive Medical Management for Preventing Recurrent Stroke in Intracranial Stenosis (SAMMPRIS), Vitesse Intracranial Stent Study for ischemic Stroke Therapy (VISSIT), Vertebral Artery Ischemia Stenting (VIST), and Vertebral Artery Stenting Trial (VAST) trial, reported a higher rate of stroke or death within 30 days in the percutaneous angioplasty and stenting (PTAS) plus best medical therapy (BMT) group (e.g., 14.7% in the SAMMPRIS trial) (1, 11–13).

Of note, approximately 24–47% of patients with acute vertebrobasilar artery occlusion have underlying ICAS and superimposed *in situ* thrombosis (14). Patients with underlying ICAS were found to have longer procedure times, lower recanalization rates, and higher rates of reocclusion than those with embolic occlusion (15–18).

Currently, there is no Level A evidence regarding the optimal rescue strategy for failed recanalization or instant/imminent reocclusion in acute ischemic stroke from large vessel occlusion due to underlying ICAS (19–21). Here, we present a case illustration of rescue therapy post intravenous tPA and thrombectomy with intra-arterial tirofiban and balloon angioplasty followed by oral dual antiplatelet therapy (DAPT) in a patient with dominant vertebral artery occlusion due to ICAS. We also performed a narrative review of the available literature on rescue therapy after thrombectomy.

## Case illustration

**Figure 1:**
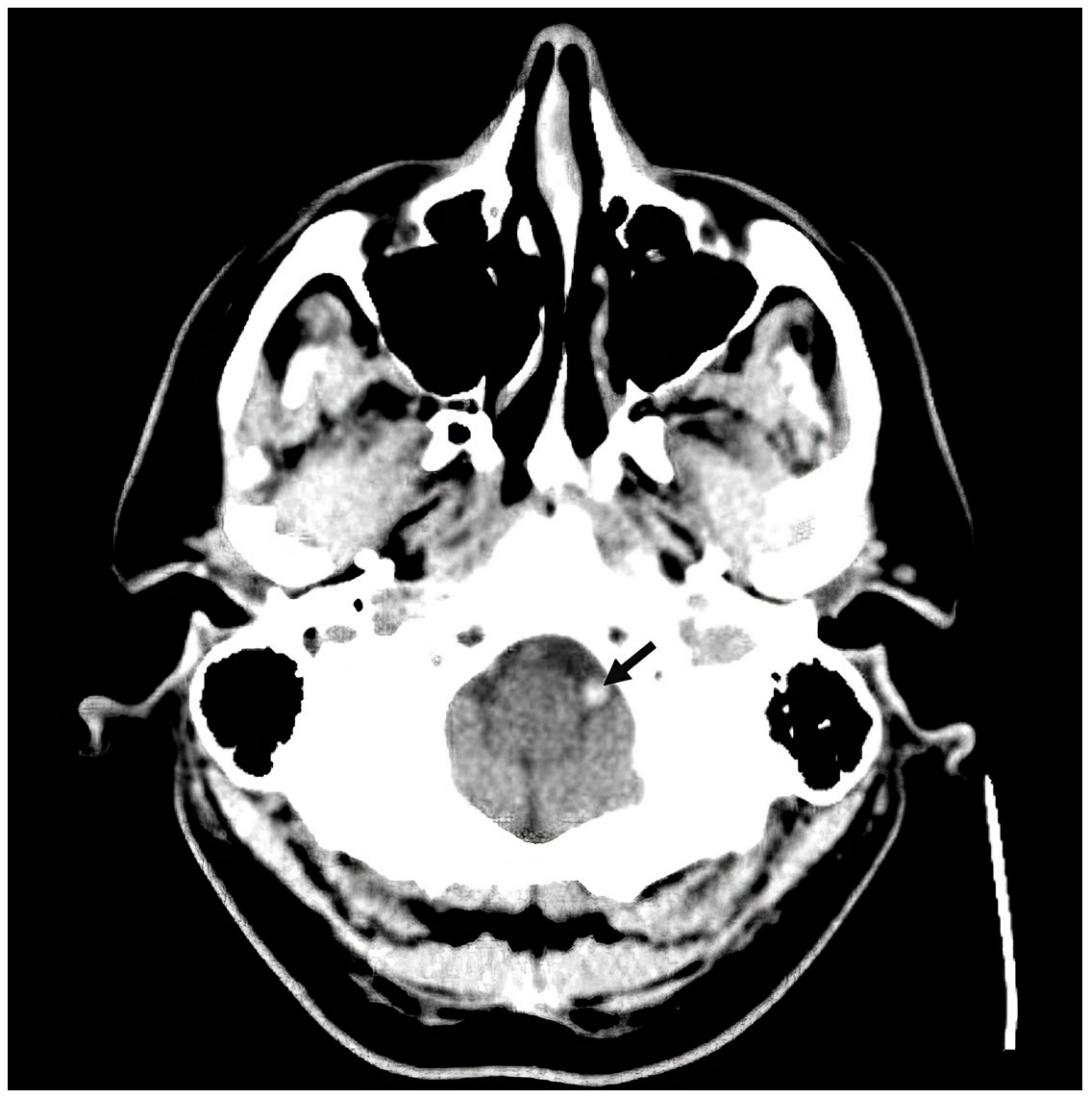
Non-contrast CT head demonstrating hyperdense vessel sign in the left intracranial vertebral artery (arrow).

**Figure 2:**
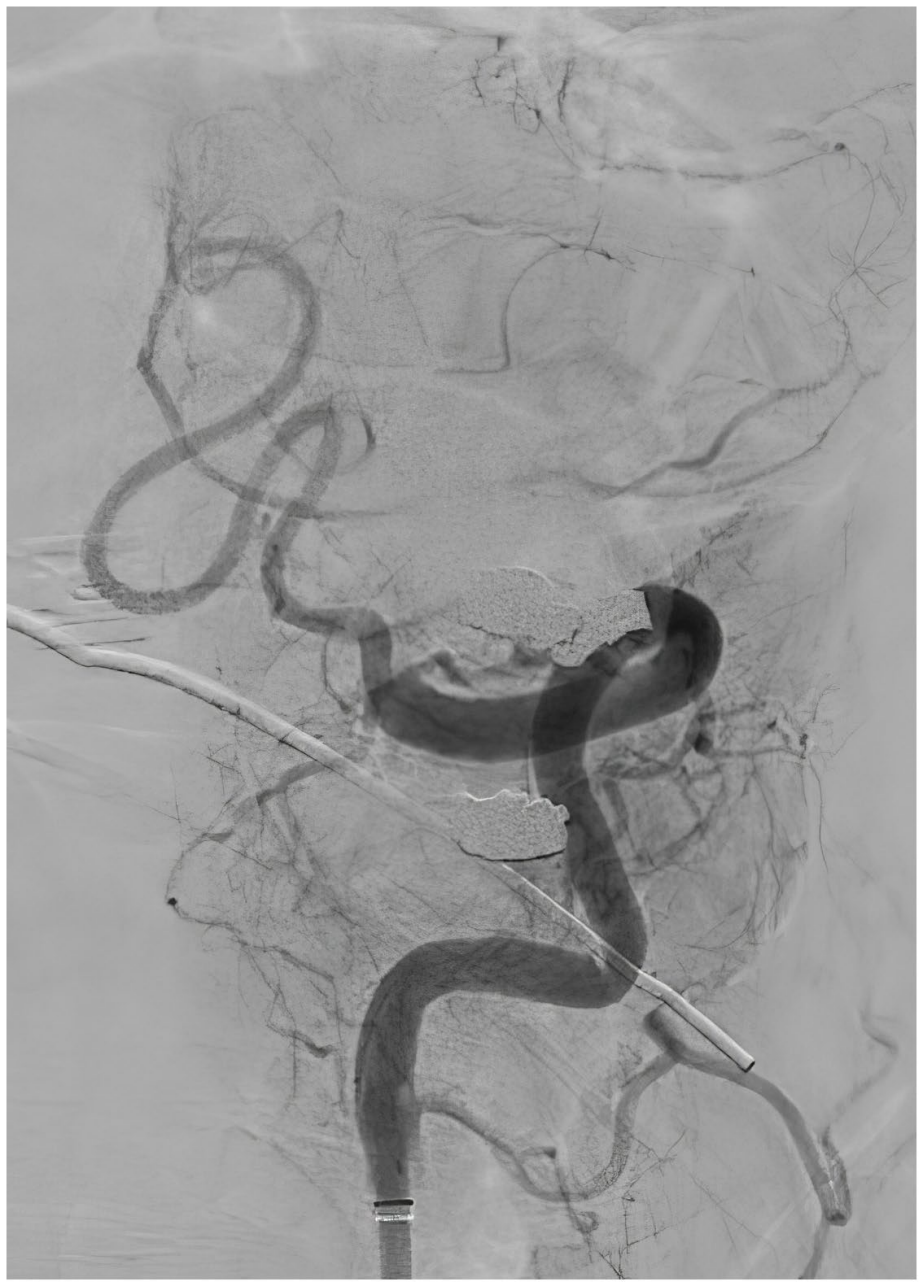
Diagnostic cerebral angiography, lateral view, demonstrating occlusion of the left vertebral artery past the level of the left posterior inferior cerebellar artery.

**Figure 3:**
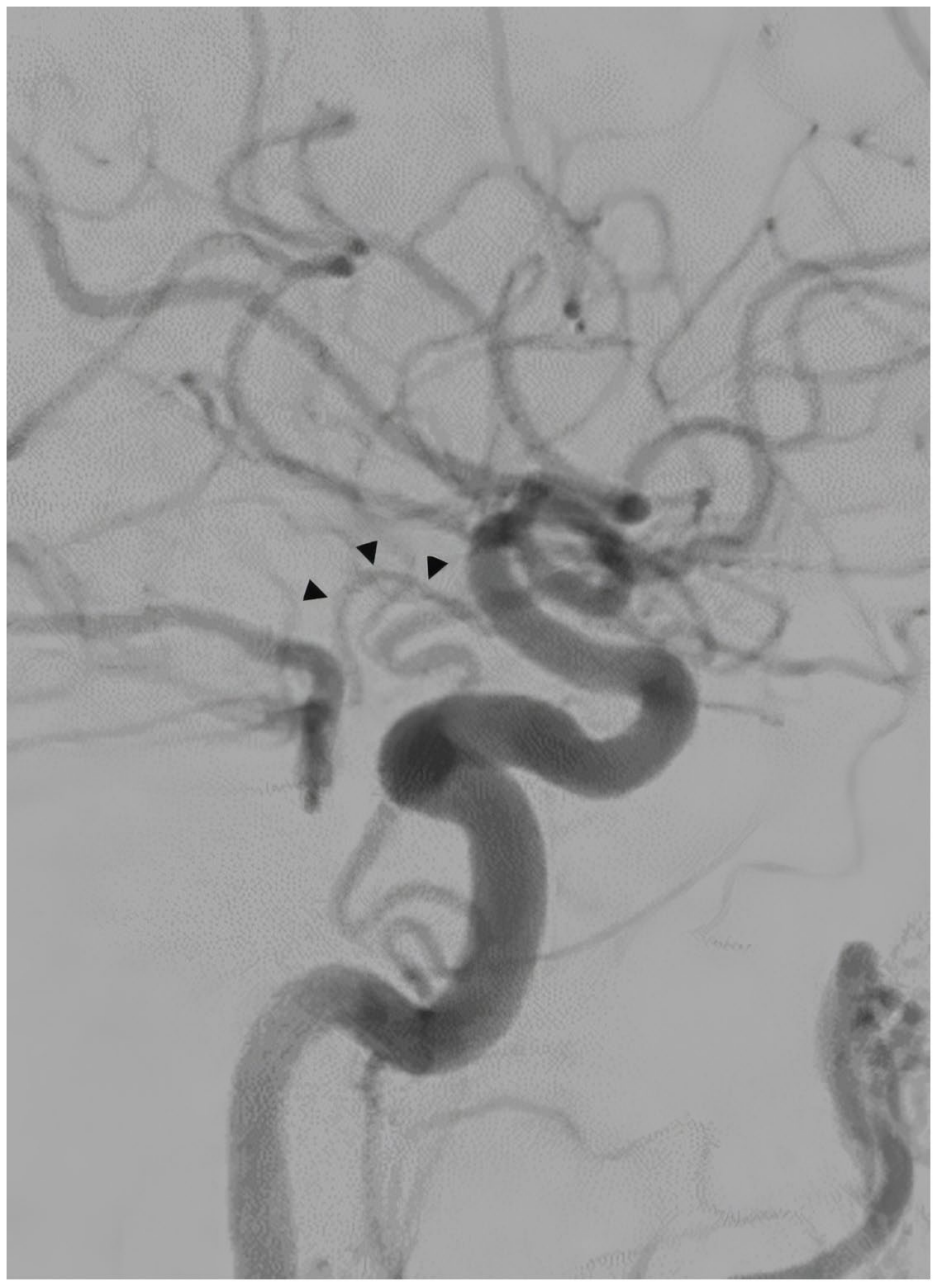
Diagnostic cerebral angiography, lateral view, distal reconstitution of the basilar and posterior cerebral arteries *via* hypoplastic right posterior communicating artery (arrowheads).

A 57-year-old man with a history of uncontrolled hypertension presented to our comprehensive stroke center 2 h after an acute onset of headache, nausea, vomiting, dysarthria, diplopia, truncal and left arm ataxia, and left face and arm numbness. His National Institutes of Health Stroke Scale (NIHSS) score was 3 for dysarthria, numbness, and arm ataxia but the patient reported that ataxia, diplopia and dysarthria are disabling. CT of the head showed no intracranial hemorrhage but a hyperdense vessel sign in the left intracranial vertebral artery (VA) (Figure 1). Intravenous tPA was administered according to the standard of stroke care. Four-vessel diagnostic cerebral angiography demonstrated that the right vertebral artery terminated in the right posterior inferior cerebellar artery (PICA) and an occlusion of the left VA past the level of the left PICA (Figure 2). There was distal reconstitution of the basilar and posterior cerebral arteries *via* hypoplastic right posterior communicating artery (Figure 3). Successful thrombectomy was performed using a stent-retriever device.

**Figure 4:**
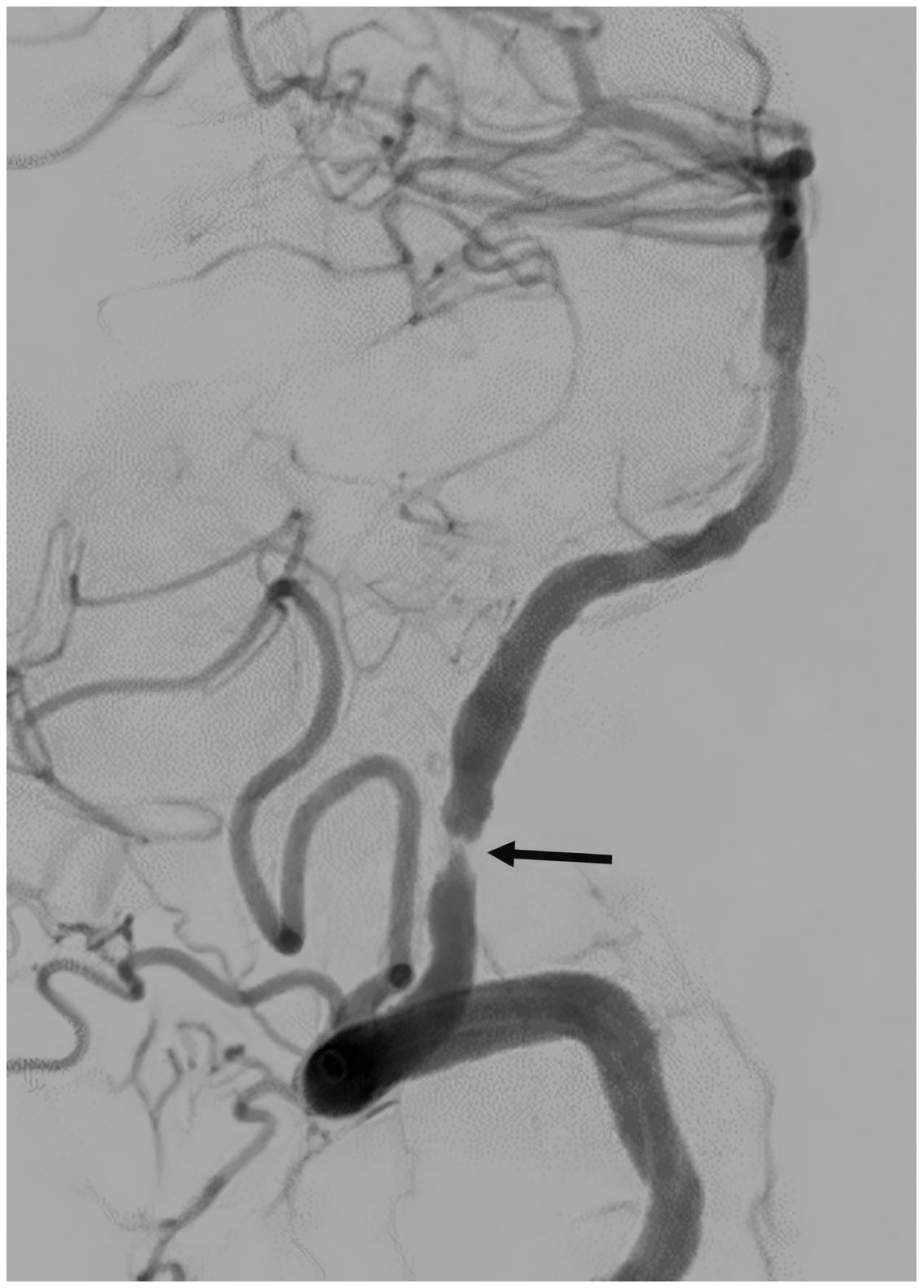
Post-thrombectomy angiography, lateral view, showing severe stenosis of the left intracranial vertebral artery (arrow).

**Figure 5:**
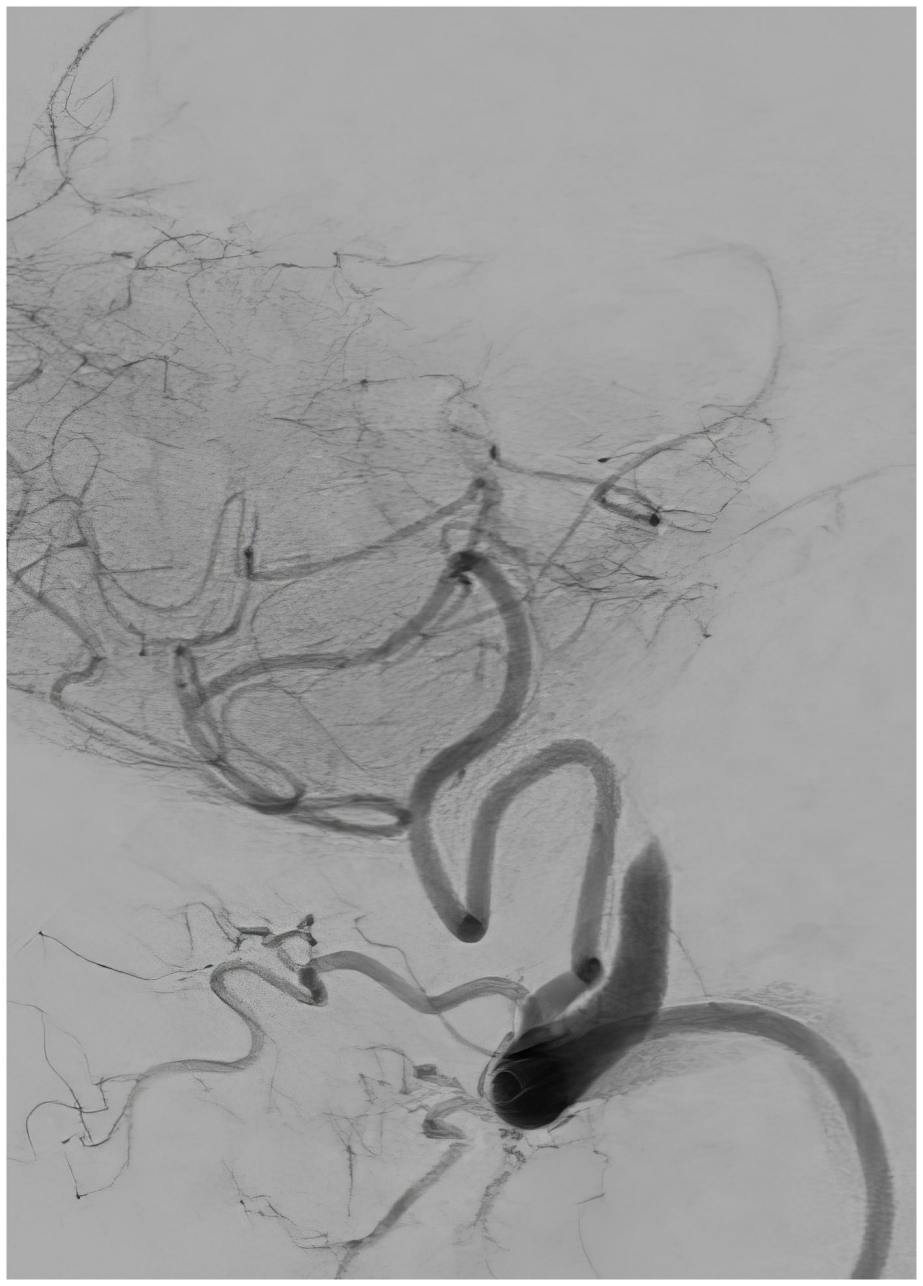
Repeat angiography, lateral view, showing reocclusion of the left vertebral artery at the level of the stenosis.

**Figure 6:**
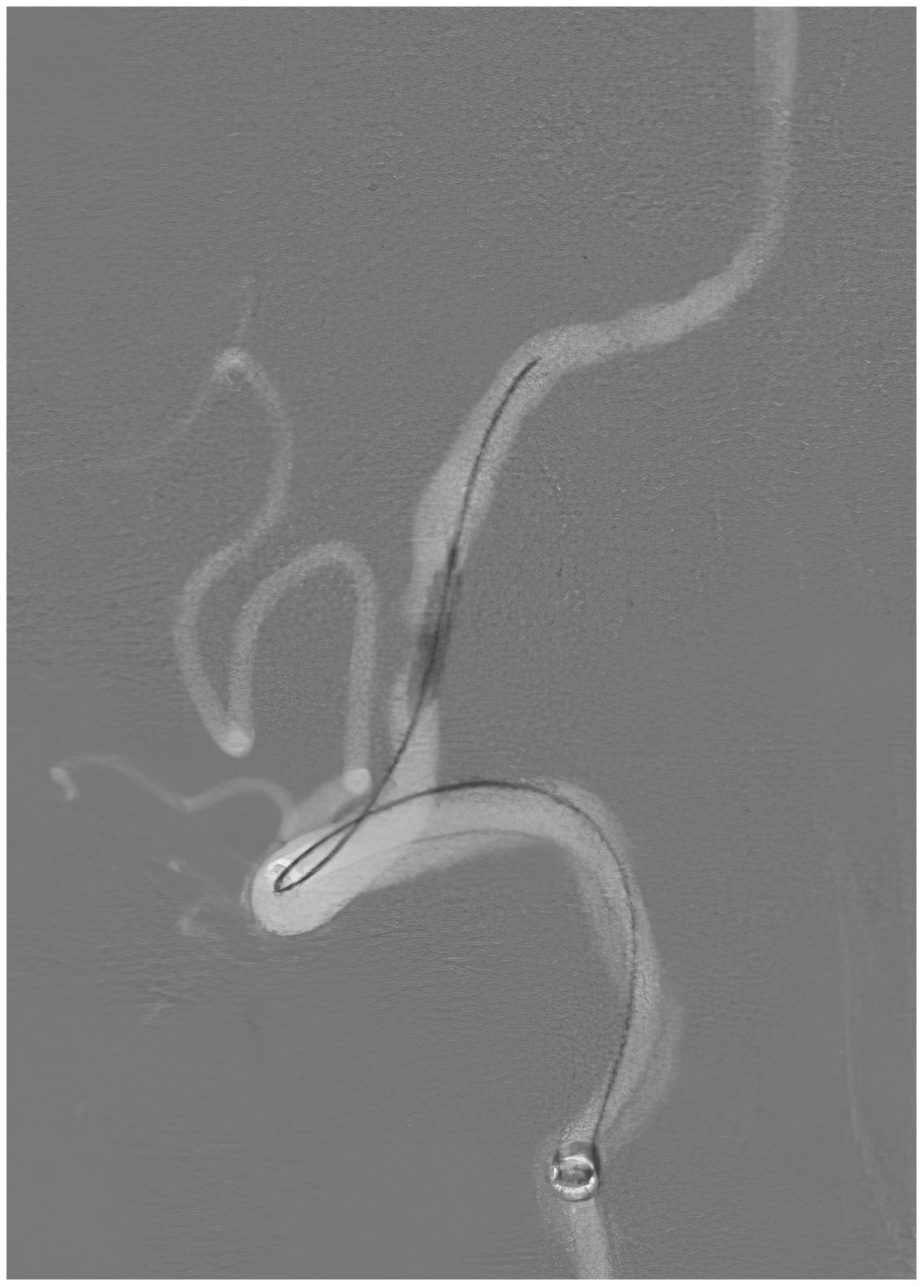
Repeat angiography, lateral view, demonstrating the balloon angioplasty of the stenotic left vertebral artery.

**Figure 7:**
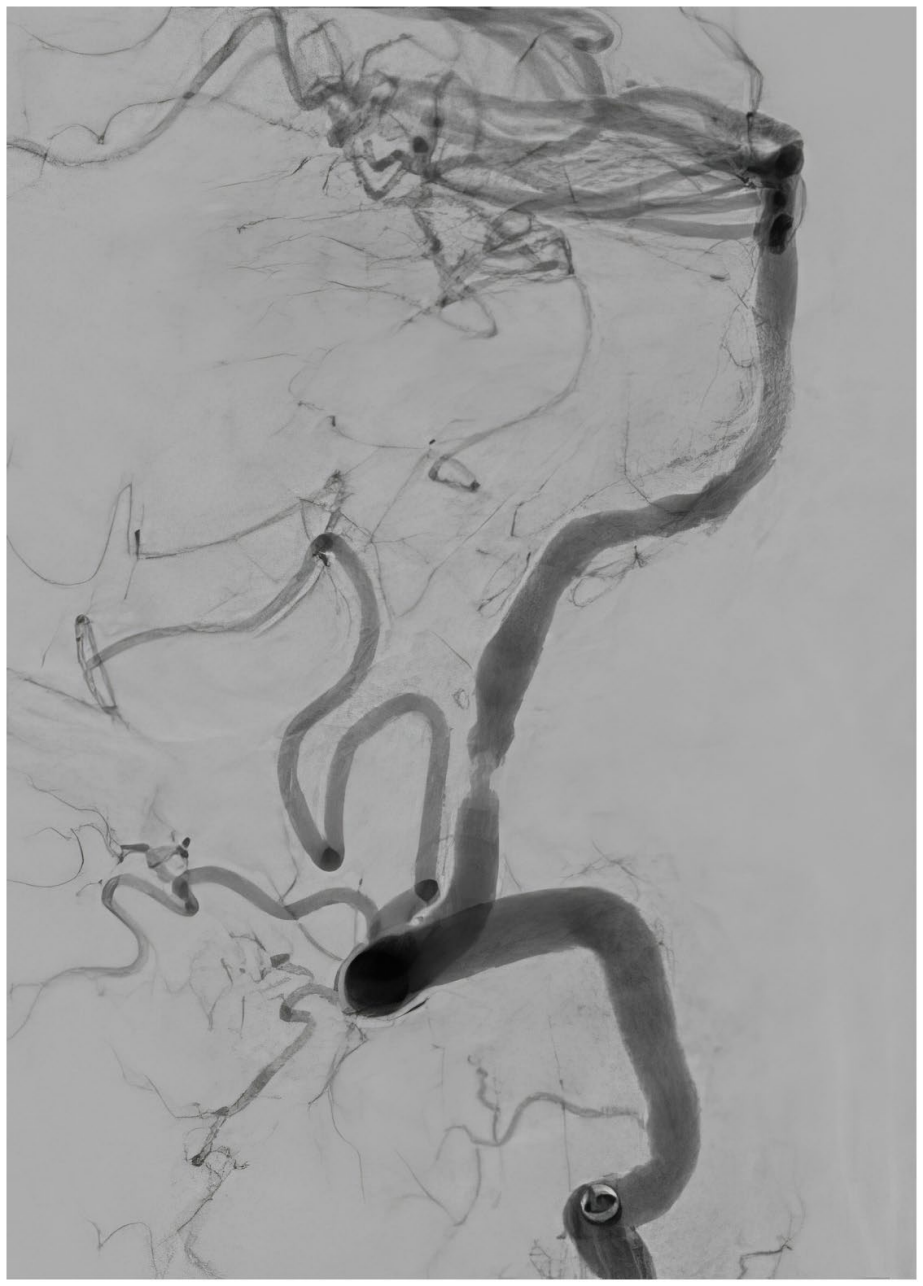
Follow-up angiography 30 min after the balloon angioplasty, lateral view, demonstrating patent left vertebral artery with residual stenosis.

**Figure 8:**
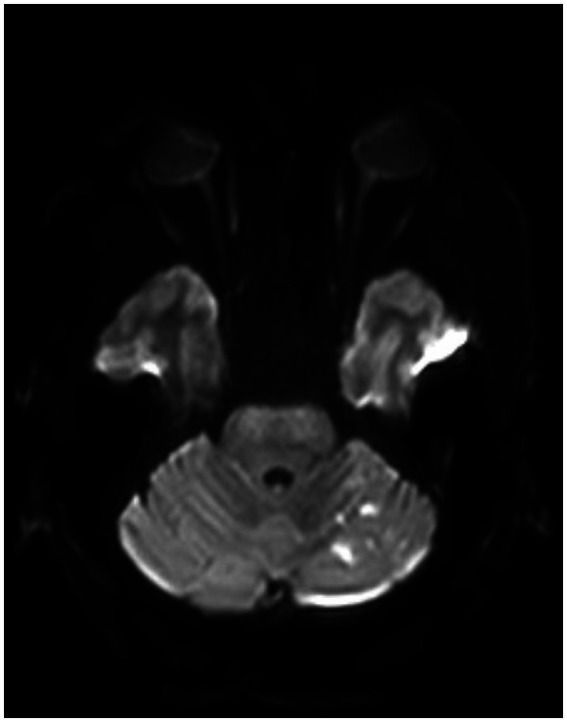
A postoperative MRI of the brain showing a low burden of scattered diffusion restriction in the bilateral cerebellar hemispheres.

**Figure 9:**
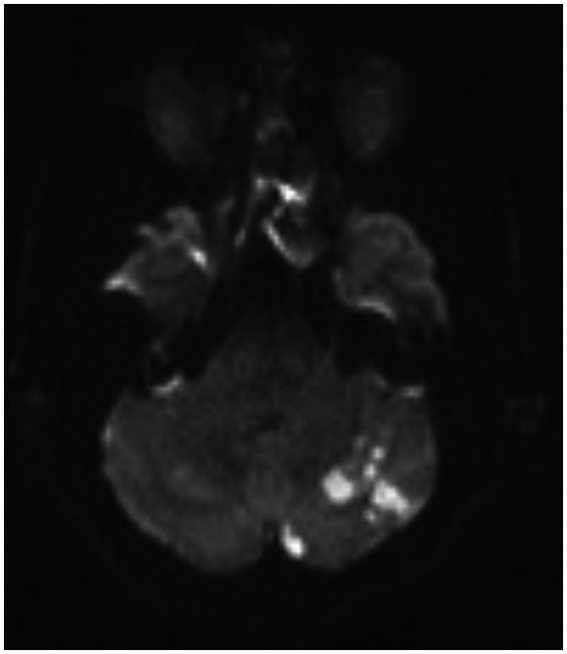
A repeat MRI of the brain showing increased burden of diffusion restriction in bilateral cerebellar hemispheres.

Immediate postretrieval angiography showed severe stenosis of the left intracranial VA (Figure 4). However, repeat angiography after 10 min showed reocclusion of the left VA at the level of the stenosis (Figure 5). A total of 250 micrograms of intra-arterial tirofiban was infused for rescue therapy followed by gentle balloon angioplasty of the stenosis (Figure 6). Repeat angiography 30 min later showed a patent left VA with improved focal stenosis (Figure 7). Given high risk of reocclusion due to ICAS and 300 mg aspirin was administered per rectum despite recent administration of tPA. The patient was started on a low-dose tirofiban infusion at a rate of 0.15 mcg/kg/min for 24 h and was bridged to dual antiplatelet therapy with 325 mg aspirin and 75 mg clopidogrel daily. The P2Y12 platelet function test result was in the therapeutic range (57 PRU) 3 days following the procedure. Postoperative MRI showed a low burden of scattered diffusion restriction in the bilateral cerebellar hemispheres (Figure 8). Follow-up MRI was obtained 2 days later due to episodes of recurrent diplopia showed an increased burden of diffusion restriction (Figure 9). Nevertheless, the patient showed remarkable recovery and had a Modified Rankin Score (mRS) of 1 upon discharge and mRS of 0 at 90 days.

## Review of literature

The efficacy of rescue therapy for failed mechanical thrombectomy or persistent severe stenosis due to underlying ICAS is not well established (15, 22, 23). The limited data mostly come from retrospective studies, including case reports, case series, registries, and cohort studies.

Additionally, the interpretation of the existing data is challenging, as most studies included a heterogeneous group of patients, such as patients with failed thrombectomy, regardless of the presence of ICAS. Furthermore, most data are from Asian patient populations due to their relatively higher predilection for developing ICAS, which makes generalizability difficult.

Acute ischemic stroke from large vessel occlusion due to ICAS is a distinct pathological process that poses unique therapeutic challenges (20). The most challenging issue is the high risk of immediate reocclusion or failure to recanalize (24). One possible explanation is that thrombectomy with a stent-retriever device may cause endothelial injury at the site of ICAS and increase thrombogenicity (25). In a case series by Forbrig et al., immediate reocclusion occurred in 25 out of 34 patients, and residual high-grade stenosis occurred in the remaining 9 (26). A meta-analysis by Tsang et al. showed that the risk of instant reocclusion is significantly higher after mechanical thrombectomy in strokes due to ICAS as compared to other types of strokes (36.9% vs. 2.7%, respectively, OR, 23.7, 95% CI 6.96–80.7) (24). In another study by Baek et al., successful permanent recanalization with stent-retriever thrombectomy was achieved in only 28.9% of occlusions caused by ICAS compared to 82.8% of occlusions due to all other etiologies (20). In two studies, comparable recanalization rates were achieved with rescue therapies such as balloon angioplasty and/or stenting, but these increased the duration of the endovascular procedure (20, 27). The latter has been shown to be an independent predictor of poor outcome (28).

Conversely, some studies have suggested that ICAS itself is an independent predictor for a good outcome, which is explained by chronic ischemic preconditioning and development of collateral circulation, resulting in a smaller core and a larger penumbra (16, 29).

Various rescue strategies have been proposed, including different combinations of dual antiplatelet therapy (DAPT), intra-arterial (IA) or intravenous (IV) infusion of glycoprotein IIb/IIIa receptor inhibitors, angioplasty and stenting (20, 21, 25, 30–32).

## Glycoprotein IIb/IIIa receptor inhibitors

Rescue therapies after failed thrombectomy include intra-arterial or intravenous bolus (dose) of GP IIb/IIIa inhibitors with or without subsequent intravenous infusion for 12–48 h, balloon angioplasty and stenting. Some of the indications are severe residual stenosis or instant reocclusion due to ICAS, failed thrombectomy, proximal thrombus with the potential to cause distal embolism or incomplete recanalization (4, 25, 33–38).

Tirofiban, eptifibatide, and abciximab are the three GP IIb/IIIa inhibitors approved for use in the United States (39). A 2021 study by Jang et al. retrospectively compared patients receiving intra-arterial tirofiban for rescue therapy to those who did not. This study included 314 patients, of whom only 35 received intra-arterial tirofiban. The authors concluded that intra-arterial tirofiban had no association with increased risk of hemorrhage, 3-month mortality or improved outcome when given for rescue therapy (36). A similar study conducted in China investigated the safety and efficacy of intra-arterial infusion of low-dose tirofiban (0.25–1 mg) followed by an intravenous infusion (0.1 mcg/kg/min) for 12–24 h in patients with underlying ICAS who needed rescue therapy for the above indications. After adjusting for confounding factors, tirofiban was associated with excellent outcomes (mRS 0) and functional independence (mRS 0–2) (adjusted OR 1.819; CI, 1.064–3.110 and OR 1.849; CI, 1.065–3.212, respectively) and was not associated with an increased risk of intracranial hemorrhage (adjusted HR 0.998; 95% CI 0.021–46.825; *p* = 0.999). The association was stronger in patients with severe stroke (NIHSS>5) and anterior circulation stroke (37).

The results of the above studies should be interpreted with caution due to the nonrandomized nature of the studies and potential selection bias.

A retrospective analysis of prospectively collected data compared standard practices between two hospitals in South Korea, one of which used angioplasty and stenting as a primary rescue therapy for failed MT in ICAS-related stroke, and the other hospital used intra-arterial infusions of 0.5–1 mg of tirofiban. There were no significant differences in the rates of successful reperfusion, symptomatic hemorrhage, 3-month modified Rankin scale score 0–2, and mortality between the two centers (28).

In general, hemorrhagic conversion in patients with posterior circulation stroke is very uncommon, and the use of tirofiban in this patient population is generally low risk (40). In addition, some authors hypothesized that patients with ICAS may be at lower risk for hemorrhagic conversion due to the formation of collaterals and ischemic preconditioning over time, resulting in a generally smaller infarct core compared to patients with other etiologies of large vessel occlusion (16, 29, 40).

### Route of administration

Currently, there is not enough evidence to support intra-arterial versus intravenous bolus of GP IIa/IIIb inhibitors, and the optimal duration of subsequent intravenous infusion is unknown (36, 38). A recent retrospective study by Yang et al. included 503 patients and compared three groups of patients who underwent mechanical thrombectomy within 24 h after stroke symptom onset. Eligible patients also received IV tPA. Most patients received no tirofiban (*n* = 354), 79 received an intraarterial bolus (10 mcg/kg) of tirofiban followed by an intravenous infusion (0.15 mcg/kg/min), and 70 received intravenous (10 mcg/kg) tirofiban followed by an intravenous infusion (0.15 mcg/kg/min). The treatment choice was at the discretion of the interventionalist. Tirofiban was infused for rescue treatment for ICAS, failed thrombectomy, proximal thrombus, or stent placement. The patients who received an intra-arterial bolus of tirofiban had a significantly higher rate of sICH (19.1% vs. 0%, *p* < 0.001), in-hospital death (23.6% vs. 0%, *p* < 0.001) and death at 3 months (26.8% vs. 11.0%, *p* = 0.021), as well as a lower rate of 90- day favorable outcome (35.4% vs. 51.2%, *p* = 0.038). In the subgroup of patients with ICAS, intravenous tirofiban increased the recanalization rate (95.3% vs. 64.1%, *p* < 0.001) and decreased the rate of poor outcome (7.0% vs. 38.6%, *p* < 0.001) compared to the nontirofiban group (38). However, the current evidence is not compelling enough to advocate for one method of tirofiban administration over another.

## Rescue balloon angioplasty without stenting

The rationale for avoiding stent placement in the setting of acute ischemic stroke includes a possible increased risk of hemorrhage with DAPT, longer procedure times and the risk of in- stent thrombosis due to a lack of standard preprocedural antiplatelet treatment (28, 30).

Therefore, balloon angioplasty alone has been proposed as an initial step for residual severe stenosis due to ICAS (23). However, some authors suggested that balloon angioplasty itself may cause further intimal injury and increase the risk of instant reocclusion, causing a “snowplowing effect” (occlusion of adjacent perforators), and may be less effective than stenting due to vessel recoiling (30).

There are only a few retrospective case series of patients who were treated primarily with balloon angioplasty for residual severe intracranial stenosis after successful mechanical thrombectomy (22, 23, 41, 42). The largest case series of 68 patients was presented by Chen et al. In that study, successful recanalization was achieved in 45 patients with balloon angioplasty alone, whereas an additional 16 patients required rescue stenting. The combined recanalization rate was 89.7% with this strategy (23).

Most recently, Ni et al. presented the results of balloon angioplasty combined with Tirofiban as a first-line rescue treatment after failed mechanical thrombectomy for middle cerebral artery occlusions and underlying ICAS. A retrospective review of 47 subjects showed an 87% (*n* = 41) rate of successful recanalization. Stent placement was attempted in the remaining 6 patients, of whom 3 had successful recanalization. Good functional outcome (mRS ≤2) was achieved in 55.3% of the patients (22).

## Rescue stenting for failed thrombectomy

Successful vessel recanalization is the key for improving the treatment outcomes of stroke (20, 34). Therefore, it is not surprising that a number of small studies have almost uniformly shown that rescue stenting for failed thrombectomy is associated with better recanalization rates and improved clinical outcomes (mRS ≤ 2) including for strokes of mild to moderate severity (NIHSS ≤9) (34, 43–47). The rate of favorable clinical outcome ranged from 35.1 to 56.5% when rescue stenting was performed versus 2.5–19.7% if the vessel was left occluded (17, 32, 34, 43, 45, 46, 48). The reported rate of successful recanalization varied widely from 59.1 to 96.5% across different studies (34, 43, 44, 46). The rate of sICH was not significantly different with either treatment modality (7.1–9.7% in patients who underwent rescue stenting vs. 10.8–14.1% in patients who were left nonrecanalized) (17, 32, 45). Stracke et al. reported higher rate of sICH after rescue stenting in anterior circulation compared to posterior circulation stroke (32). They also reported lower rates of sICH with the use of the Acclino/Acclinoflex stent (Acandis GmbH) compared to other stents (3.3% vs. 14.3%; *p* < 0.01) (32, 49).

A few studies found a lower mortality rate in patients with rescue stenting (15–28% vs. 46.5–50%) (45, 48). However, one study, which analyzed data from 53 patients, reported no statistically significant difference in mortality rates (43).

The largest study evaluating rescue intracranial stenting for failed thrombectomy was a retrospective review of a prospectively collected database of 499 patients in the SAINT (Stenting and Angioplasty in Neurothrombectomy) Study. Rescue intracranial stenting was compared to failed recanalization (modified Thrombolysis in Cerebral Ischemia score 0–1).

Compared with the failed reperfusion group, rescue intracranial stenting had a favorable shift in the overall mRS score distribution (acOR, 2.31 [95% CI, 1.61–3.32]; *p* < 0.001), higher rates of functional independence (35.1% vs. 7%; adjusted odds ratio [aOR], 6.33 [95% CI, 3.14–12.76]; *p* < 0.001), and lower mortality (28% vs. 46.5%; aOR, 0.55 [95% CI, 0.31–0.96]; *p* = 0.04) at 90 days. The rates of sICH were comparable across both groups (7.1% vs. 10.2%; aOR, 0.99 [95% CI, 0.42–2.34]; *p* = 0.98), even after a matched cohort analysis (45).

Additionally, a meta-analysis of pooled data including 530 patients, of whom 365 underwent stenting, showed that rescue intracranial stenting after failed mechanical thrombectomy or high failure risk thrombectomy results in improved clinical outcomes compared with patients without stenting (48.5% vs. 19.7%, respectively; *p* < 0.001), without an increase in the rate of sICH, despite any additional use of antiplatelet agents (9.7% vs. 14.1%, respectively; *p* = 0.04) (17).

Two studies showed no significant improvement with rescue intracranial stenting for a failed thrombectomy. Hassan et al. conducted a nonrandomized study involving 420 patients with stroke due to underlying ICAS who failed mechanical thrombectomy (TICI 0-2A). Forty-six patients underwent emergency stenting, whereas the remaining 374 patients were treated medically. Acute intracranial stenting in addition to mechanical thrombectomy was not associated with an increase in overall length of stay, intracerebral hemorrhage rates, or any change in discharge mRS score (18).

Zhou et al. conducted a study involving 68 patients with failed thrombectomy, of whom 47 received rescue stent placement. The rate of successful recanalization was 80.85%, and a favorable outcome was achieved in 57.45% of the patients at 90 days. Of note, in the stenting group, only 2 patients (4.26%) were reported to have atrial fibrillation as the possible etiology of stroke. The time from groin puncture to recanalization was significantly longer in the stenting group (*p* = 0.03), and there was no difference in the rate of intracranial hemorrhage (50).

One study investigated the outcomes of rescue intracranial stenting in failed thrombectomy of the basilar artery. Luo et al. performed a subgroup analysis of data from the Endovascular Treatment Key Technique and Emergency Work Flow Improvement of Acute.

Ischemic Stroke (ANGEL-ACT) prospective registry in China. Among the 93 patients who failed thrombectomy, 81 (87.1%) received rescue stenting with a 92.6% recanalization rate.

Compared with the patients who did not receive rescue therapy (*n* = 12), the patients who underwent rescue stenting had a higher rate of favorable clinical outcomes (modified Rankin Scale score at 90 days postprocedure, 0–3: 16.7 vs. 51.9%, respectively; *p* = 0.023) without an increase in the rate of sICH, but with a significantly lower mortality rate (58.3 vs. 18.5%; *p* = 0.006) (51). Some of the reported independent predictors of poor functional outcome after rescue intracranial stenting for failed thrombectomy are high NIHSS score upon (aOR 1.10; *p* = 0.002), a higher pre-existing mRS (aOR 2.02; *p* = 0.049), and a modified Thrombolysis in Cerebral Infarction score 0 to 2a following stenting (aOR 23.24; *p* < 0.001) (32).

## Emergent stenting of ICAS following successful thrombectomy

There is currently no strong evidence to support emergent stenting after successful thrombectomy, as the data are scarce and limited to case series and cohort studies (26, 27, 52–56). Li et al. performed a comparative analysis of 184 consecutive patients with severe stenosis after thrombectomy, in which 64 patients underwent rescue angioplasty or stenting, and 120 patients were managed medically. Intracranial angioplasty/stenting resulted in better functional outcomes (51.6% vs. 35.0%, *p =* 0.02) and a lower 24-h reocclusion rate (6.3% vs. 17.5%, *p* = 0.03). All patients had stroke in the anterior circulation (52). In another multicenter prospective cohort study enrolling a total of 113 consecutive patients with underlying ICAS >70% in the anterior cerebral circulation, 81 (71.7%) received emergent angioplasty and/or stenting after thrombectomy. The patients in the emergent angioplasty and/or stenting group were significantly more likely to have recanalization at 24 h (adjusted OR [aOR], 3.782; 95% confidence interval [CI], 1.821–9.125; *p* = 0.02) and less likely to have early neurologic deterioration (aOR, 0.299; 95% CI, 0.110–0.821; *p* = 0.01) (55). There was no significant increase in sICH (aOR, 0.710; 95% CI, 0.199–2.622; *p* = 0.67), death (aOR, 0.581; 95% CI, 0.186–2.314; *p* = 0.41), or functional independence at 90 days (aOR, 1.752; 95% CI, 0.774–3.257; *p* = 0.16) (55).

In a meta-analysis of 1,315 subjects, 261 underwent emergent intracranial stenting for residual stenosis after thrombectomy. The pooled estimate of the successful recanalization rate was 88% (95% CI 84–92%), and the rate of favorable outcomes was 52% (95% CI, 47–56%).

Symptomatic intracranial hemorrhage occurred in 5% of the patients, and the mortality rate was 15% (54).

One study performed a comparative analysis of outcomes between Tirofiban infusion alone and rescue angioplasty/stenting as a primary treatment strategy for underlying severe ICAS after mechanical thrombectomy (57). Two comprehensive stroke centers prospectively collected data on 140 consecutive patients. There were no significant differences between the two centers in the rate of successful reperfusion, parenchymal hemorrhage, sICH, 3-month mRS score, and mortality (57).

## Conclusion

- Tirofiban may be beneficial for rescue therapy after failed thrombectomy or residual severe intracranial stenosis.
- Balloon angioplasty and/or stenting may be beneficial as a rescue treatment for failed thrombectomy or impending reocclusion. The role of immediate stenting for residual stenosis after successful thrombectomy is still unclear, and randomized clinical trials are needed.
- Rescue treatment with GPIIa/IIIb receptor inhibitors and/or balloon angioplasty and/or stenting does not appear to significantly increase the risk of symptomatic intracranial hemorrhage.

## Author contributions

TK, MS, HA, JS, MN, and JG: conceived and designed the review, searched and selected relevant articles, extracted data, synthesized the findings, and wrote the manuscript. IY, KG, SS, and WY: critically reviewed the article, provided scientific expertise and assisted in the interpretation of the findings. All authors contributed to the article and approved the submitted version.

## Conflict of interest

The authors declare that the research was conducted in the absence of any commercial or financial relationships that could be construed as a potential conflict of interest.

## Publisher’s note

All claims expressed in this article are solely those of the authors and do not necessarily represent those of their affiliated organizations, or those of the publisher, the editors and the reviewers. Any product that may be evaluated in this article, or claim that may be made by its manufacturer, is not guaranteed or endorsed by the publisher.

## References

[1] Chimowitz MI Lynn MJ Derdeyn CP Turan TN Fiorella D Lane BF. Stenting versus aggressive medical therapy for intracranial arterial stenosis. N Engl J Med. (2011) 365:993–1003. doi: 10.1056/NEJMoa1105335, PMID: 21899409 PMC3552515

[2] Gorelick PB Wong KS Bae HJ Pandey DK. Large artery intracranial occlusive disease: a large worldwide burden but a relatively neglected frontier. Stroke. (2008) 39:2396–9. doi: 10.1161/STROKEAHA.107.505776, PMID: 18535283

[3] Wong LKS. Global burden of intracranial atherosclerosis. Int J Stroke. (2006) 1:158–9. doi: 10.1111/j.1747-4949.2006.00045.x, PMID: 18706036

[4] Kang DH Kim YWSW Hwang YH Park SP Kim YWSW Baik SK. Instant reocclusion following mechanical thrombectomy of in situ thromboocclusion and the role of low-dose intra-arterial tirofiban. Cerebrovasc Dis. (2014) 37:350–5. doi: 10.1159/000362435 24941966

[5] Derdeyn CP Chimowitz MI Lynn MJ Fiorella D Turan TN Janis LS. Aggressive medical treatment with or without stenting in high-risk patients with intracranial artery stenosis (SAMMPRIS): The final results of a randomised trial. Lancet. (2014) 383:333–41. doi: 10.1016/S0140-6736(13)62038-3 24168957 PMC3971471

[6] Lutsep HL Barnwell SL Larsen DT Lynn MJ Hong M Turan TN. Outcome in patients previously on antithrombotic therapy in the SAMMPRIS trial: subgroup analysis. Stroke. (2015) 46:775–9. doi: 10.1161/STROKEAHA.114.007752, PMID: 25593135 PMC4342339

[7] Lutsep HL Lynn MJ Cotsonis GA Derdeyn CP Turan TN Fiorella D. Does the stenting versus aggressive medical therapy trial support stenting for subgroups with intracranial stenosis? Stroke. (2015) 46:3282–4. doi: 10.1161/STROKEAHA.115.009846, PMID: 26382173 PMC4624506

[8] Gao P Wang T Wang D Liebeskind DS Shi H Li T. Effect of stenting plus medical therapy vs medical therapy alone on risk of stroke and death in patients with symptomatic intracranial stenosis: the CASSISS randomized clinical trial. JAMA. (2022) 328:534–42. doi: 10.1001/jama.2022.12000, PMID: 35943472 PMC9364128

[9] Kleindorfer DO Towfighi A Chaturvedi S Cockroft KM Gutierrez J Lombardi-Hill D. Guideline for the prevention of stroke in patients with stroke and transient ischemic attack: a guideline from the American Heart Association/American Stroke Association. Stroke. (2021, 2021) 52:E364–467. doi: 10.1161/STR.0000000000000375 34024117

[10] Padalia A Sambursky JA Skinner C Moureiden M. Percutaneous transluminal angioplasty with stent placement versus best medical therapy alone in symptomatic intracranial arterial stenosis: a best evidence review. Cureus. (2018) 10:e2988. doi: 10.7759/cureus.2988, PMID: 30397562 PMC6207274

[11] Compter A Van der Worp HB Schonewille WJ Vos JA Algra A Lo TH. VAST: vertebral artery stenting trial. Protocol for a randomised safety and feasibility trial. Trials. (2008) 9:1–8. doi: 10.1186/1745-6215-9-65 19025615 PMC2611963

[12] Zaidat OO Fitzsimmons BF Woodward BK Wang Z Killer-Oberpfalzer M Wakhloo A. Effect of a balloon-expandable intracranial stent vs medical therapy on risk of stroke in patients with symptomatic intracranial stenosis: the VISSIT randomized clinical trial. JAMA. (2015) 313:1240–8. doi: 10.1001/jama.2015.1693, PMID: 25803346

[13] Markus HS Larsson SC Kuker W Schulz UG Ford I Rothwell PM. Stenting for symptomatic vertebral artery stenosis: the vertebral artery Ischaemia stenting trial. Neurol Int. (2017) 89:1229–36. doi: 10.1212/WNL.0000000000004385, PMID: 28835400 PMC5606920

[14] Yu W Higashida RT. Endovascular Thrombectomy for acute basilar artery occlusion: latest findings and critical thinking on future study design. Transl Stroke Res. (2022) 13:913–22. doi: 10.1007/s12975-022-01008-5, PMID: 35349051 PMC9613579

[15] Kang DH Yoon W. Current opinion on endovascular therapy for emergent large vessel occlusion due to underlying intracranial atherosclerotic stenosis. Korean J Radiol. (2019) 20:739–48. doi: 10.3348/kjr.2018.0809, PMID: 30993925 PMC6470088

[16] Luo G Mo D Tong X Liebeskind DS Song L Ma N. Factors associated with 90-day outcomes of patients with acute posterior circulation stroke treated by mechanical Thrombectomy. World Neurosurg. (2018) 109:e318–28. doi: 10.1016/j.wneu.2017.09.171, PMID: 28987852

[17] Maingard J Phan K Lamanna A Kok HK Barras CD Russell J. Rescue intracranial stenting after failed mechanical Thrombectomy for acute ischemic stroke: a systematic review and Meta-analysis. World Neurosurg. (2019) 132:e235–45. doi: 10.1016/j.wneu.2019.08.192, PMID: 31493593

[18] Hassan AE Ringheanu VM Preston L Tekle WG Qureshi AI. Acute intracranial stenting with mechanical thrombectomy is safe and efficacious in patients diagnosed with underlying intracranial atherosclerotic disease. Interv Neuroradiol. (2022) 28:419–25. doi: 10.1177/15910199211039403, PMID: 34515574 PMC9326867

[19] Al Kasab S Almallouhi E Spiotta AM. Rescue endovascular treatment for emergent large vessel occlusion with underlying intracranial atherosclerosis: current state and future directions. Front Neurol. (2021) 12:734971. doi: 10.3389/fneur.2021.734971 34759882 PMC8573125

[20] Baek JH Kim BM Heo JH Kim DJ Nam HS Kim YD. Outcomes of endovascular treatment for acute intracranial atherosclerosis-related large vessel occlusion. Stroke. (2018) 49:2699–705. doi: 10.1161/STROKEAHA.118.022327, PMID: 30355204

[21] Wu Y Wang J Sun R Feng G Li W Gui Y. A novel endovascular therapy strategy for acute ischemic stroke due to intracranial atherosclerosis-related large vessel occlusion: stent-pass-aspiration-resCuE-Micowire-angioplasty (SPACEMAN) technique. Front Neurol. (2022) 13:798542. doi: 10.3389/fneur.2022.798542 35237229 PMC8882581

[22] Ni H Hang Y Wang CD Liu S Jia ZY Bin SH. Balloon angioplasty combined with tirofiban as a first-line rescue treatment after failed mechanical thrombectomy for middle cerebral artery occlusion with underlying atherosclerosis. World Neurosurg. (2022) 166:e306–12. doi: 10.1016/j.wneu.2022.07.001, PMID: 35809841

[23] Chen W Gong J Song R Liu J Wang M Zhang T. Efficacy and safety of direct balloon angioplasty in the treatment of large atherosclerotic stroke. Clin Neurol Neurosurg. (2021) 211:107035. doi: 10.1016/j.clineuro.2021.107035, PMID: 34826756

[24] Tsang ACO Orru E Klostranec JM Yang IH Lau KK Tsang FCP. Thrombectomy outcomes of intracranial atherosclerosis-related occlusions: a systematic review and meta-analysis. Stroke. (2019) 50:1460–6. doi: 10.1161/STROKEAHA.119.024889, PMID: 31084327

[25] Kim YSW Il SS Yoo J JHJM H Kim CH Kang DH. Local tirofiban infusion for remnant stenosis in large vessel occlusion: Tirofiban ASSIST study. BMC Neurol. (2020) 20:284. doi: 10.1186/s12883-020-01864-4 32689957 PMC7370431

[26] Forbrig R Lockau H Flottmann F Boeckh-Behrens T Kabbasch C Patzig M. Intracranial rescue stent angioplasty after stent-retriever thrombectomy: multicenter experience. Clin Neuroradiol. (2019) 29:445–57. doi: 10.1007/s00062-018-0690-4 29761219

[27] Dobrocky T Kaesmacher J Bellwald S Piechowiak E Mosimann PJ Zibold F. Stent-retriever thrombectomy and rescue treatment of M1 occlusions due to underlying intracranial atherosclerotic stenosis: cohort analysis and review of the literature. Cardiovasc Intervent Radiol. (2019) 42:863–72. doi: 10.1007/s00270-019-02187-9 30859286

[28] Kang DH Kim YW Hwang YH Kim YS. Endovascular recanalization of acute tandem cervical carotid and intracranial occlusions: efficacy of cervical balloon angioplasty alone then intracranial target recanalization strategy. World Neurosurg. (2019) 126:e1268–75. doi: 10.1016/j.wneu.2019.02.240, PMID: 30898749

[29] Yoon W Kim SK Park MS Kim BC Kang HK. Endovascular treatment and the outcomes of atherosclerotic intracranial stenosis in patients with hyperacute stroke. Neurosurgery. (2015) 76:680–6. doi: 10.1227/NEU.0000000000000694, PMID: 25988927

[30] Lee JS Lee S-J Hong JM Alverne FJAM Lima FO Nogueira RG. Endovascular treatment of large vessel occlusion strokes due to intracranial atherosclerotic disease. J Stroke. (2022) 24:3–20. doi: 10.5853/jos.2021.01375, PMID: 35135056 PMC8829471

[31] Abou-Chebl A Bajzer CT Krieger DW Furlan AJ Yadav JS. Multimodal therapy for the treatment of severe ischemic stroke combining GPIIb/IIIa antagonists and angioplasty after failure of thrombolysis. Stroke. (2005) 36:2286–8. doi: 10.1161/01.STR.0000179043.73314.4f, PMID: 16179581

[32] Stracke CP Fiehler J Meyer L Thomalla G Krause LU Lowens S. Emergency intracranial stenting in acute stroke: predictors for poor outcome and for complications. J Am Heart Assoc. (2020) 9:e012795. doi: 10.1161/JAHA.119.012795, PMID: 32122218 PMC7335566

[33] Baik SK Oh SJ Park KP Lee JH. Intra-arterial tirofiban infusion for partial recanalization with stagnant flow in hyperacute cerebral ischemic stroke. Interv Neuroradiol. (2011) 17:442–51. doi: 10.1177/159101991101700408, PMID: 22192548 PMC3296504

[34] Baracchini C Farina F Soso M Viaro F Favaretto S Palmieri A. Stentriever thrombectomy failure: a challenge in stroke management. World Neurosurg. (2017) 103:57–64. doi: 10.1016/j.wneu.2017.03.070, PMID: 28347898

[35] Yan Z Shi Z Wang Y Zhang C Cao J Ding C. Efficacy and safety of low-dose Tirofiban for acute intracranial atherosclerotic stenosis related occlusion with residual stenosis after endovascular treatment. J Stroke Cerebrovasc Dis. (2020) 29:104619. doi: 10.1016/j.jstrokecerebrovasdis.2019.104619, PMID: 31982305

[36] Jang SH Sohn SI Park H Lee SJ Kim YW Hong JM. The safety of intra-arterial tirofiban during endovascular therapy after intravenous thrombolysis. Am J Neuroradiol. (2021) 42:1633–7. doi: 10.3174/ajnr.A7203, PMID: 34301637 PMC8423052

[37] Huo X Raynald Wang A Mo D Gao F Ma N. Safety and efficacy of Tirofiban for acute ischemic stroke patients with large artery atherosclerosis stroke etiology undergoing endovascular therapy. Front Neurol. (2021) 12:630301. doi: 10.3389/fneur.2021.630301 33643207 PMC7905208

[38] Yang J Wu Y Gao X Bivard A Levi CR Parsons MW. Intraarterial versus intravenous tirofiban as an adjunct to endovascular thrombectomy for acute ischemic stroke. Stroke. (2020) 51:2925–33. doi: 10.1161/STROKEAHA.120.029994, PMID: 32933416

[39] King S Short M Harmon C. Glycoprotein IIb/IIIa inhibitors: the resurgence of tirofiban. Vasc Pharmacol. (2016) 78:10–6. doi: 10.1016/j.vph.2015.07.008, PMID: 26187354

[40] Yu W Liu L. Therapeutic window beyond cerebral ischemic reperfusion injury BT In: Jiang W Yu W Qu Y Shi Z Luo B Zhang JH, editors. Cerebral Ischemic Reperfusion Injuries (CIRI): Bench Research and Clinical Implications. Cham: Springer International Publishing (2018). 245–59.

[41] Zhang G Ling Y Zhu S Wu P Wang C Qi J. Direct angioplasty for acute ischemic stroke due to intracranial atherosclerotic stenosis-related large vessel occlusion. Interv Neuroradiol. (2020) 26:602–7. doi: 10.1177/1591019920949674 32777960 PMC7645183

[42] Yi TY Chen WH Wu YM Zhang MF Chen YH Wu ZZ. Special endovascular treatment for acute large artery occlusion resulting from atherosclerotic disease. World Neurosurg. (2017) 103:65–72. doi: 10.1016/j.wneu.2017.03.108, PMID: 28377257

[43] Kim JH Choi J-I. Feasibility of rescue stenting technique in patients with acute ischemic stroke due to middle cerebral artery occlusion after failed thrombectomy: a single-center retrospective experience. PLoS One. (2022) 17:e0274842. doi: 10.1371/journal.pone.0274842, PMID: 36166451 PMC9514649

[44] Chang Y Kim BM Bang OY Baek JH Heo JH Nam HS. Rescue stenting for failed mechanical thrombectomy in acute ischemic stroke a multicenter experience. Stroke. (2018) 49:958–64. doi: 10.1161/STROKEAHA.117.020072, PMID: 29581342

[45] Mohammaden MH Haussen DC Al-Bayati AR Hassan A Tekle W Fifi J. Stenting and angioplasty in neurothrombectomy: matched analysis of rescue intracranial stenting versus failed Thrombectomy. Stroke. (2022) 53:2779–88. doi: 10.1161/STROKEAHA.121.038248, PMID: 35770672

[46] Tran C-C Le M-T Baxter B-W Nguyen-Luu G Ngo M-T Nguyen-Dao N-H. Rescue intracranial stenting in acute ischemic stroke: a preliminary Vietnamese study. Eur Rev Med Pharmacol Sci. (2022) 26:6944–52. doi: 10.26355/eurrev_202210_29875, PMID: 36263574

[47] Meyer L Fiehler J Thomalla G Krause LU Lowens S Rothaupt J. Intracranial stenting after failed thrombectomy in patients with moderately severe stroke: a multicenter cohort study. Front Neurol. (2020) 11:593. doi: 10.3389/fneur.2020.00593, PMID: 32117041 PMC7034674

[48] Pérez-García C Gómez-Escalonilla C Rosati S López-Ibor L Egido JA Simal P. Use of intracranial stent as rescue therapy after mechanical thrombectomy failure-9-year experience in a comprehensive stroke Centre. Neuroradiology. (2020) 62:1475–83. doi: 10.1007/s00234-020-02487-9, PMID: 32607747

[49] Stracke CP Meyer L Fiehler J Leischner H Bester M Buhk JH. Intracranial bailout stenting with the Acclino (flex) stent/NeuroSpeed balloon catheter after failed thrombectomy in acute ischemic stroke: a multicenter experience. J Neurointerv Surg. (2020) 12:43–7. doi: 10.1136/neurintsurg-2019-014957 31239330

[50] Zhou T Li T Zhu L Wang M He Y Shao Q. Intracranial stenting as a rescue therapy for acute ischemic stroke after stentriever thrombectomy failure. World Neurosurg. (2018) 120:e181–7. doi: 10.1016/j.wneu.2018.08.002, PMID: 30099177

[51] Luo G Gao F Zhang X Jia B Huo X Liu R. Intracranial stenting as rescue therapy after failure of mechanical Thrombectomy for basilar artery occlusion: data from the ANGEL-ACT registry. Front Neurol. (2021) 12:739213. doi: 10.3389/fneur.2021.739213 34659098 PMC8514631

[52] Li W Sui X Li C Zhao W Yuan S Dou S. Emergency angioplasty or stenting for stroke patients with intracranial atherosclerotic large vessel occlusion. J Atheroscler Thromb. (2023) 30:160–9. doi: 10.5551/jat.63381, PMID: 35466122 PMC9925205

[53] Potts MB da Matta L Abdalla RN Shaibani A Ansari SA Jahromi BS. Stenting of Mobile calcified emboli after failed Thrombectomy in acute ischemic stroke: case report and literature review. World Neurosurg. (2020) 135:245–51. doi: 10.1016/j.wneu.2019.12.096, PMID: 31881346

[54] Li H Zhang YY Zhang L Li Z Xing P Zhang YY. Endovascular treatment of acute ischemic stroke due to intracranial atherosclerotic large vessel occlusion: a systematic review. Clin Neuroradiol. (2020) 30:777–87. doi: 10.1007/s00062-019-00839-4, PMID: 31616958

[55] Wu C Chang W Wu D Wen C Zhang J Xu R. Angioplasty and/or stenting after thrombectomy in patients with underlying intracranial atherosclerotic stenosis. Neuroradiology. (2019) 61:1073–81. doi: 10.1007/s00234-019-02262-5, PMID: 31353425

[56] Machado M Borges de Almeida G Sequeira M Pedro F Fior A Carvalho R. Percutaneous transluminal angioplasty and stenting in acute stroke caused by basilar artery steno-occlusive disease: the experience of a single stroke Centre. Interv Neuroradiol. (2022) 28:547–55. doi: 10.1177/15910199211051830, PMID: 34704502 PMC9511620

[57] Kang D-H Yoon W Kim SK Baek BH Lee YY Kim Y-SY-WY-S. Endovascular treatment for emergent large vessel occlusion due to severe intracranial atherosclerotic stenosis. J Neurosurg. (2018) 1306:1–8. doi: 10.3171/2018.1.JNS172350 29932374

